# Academy of Stomatology

**Published:** 1899-02

**Authors:** 

**Affiliations:** Academy of Stomatology


### ACADEMY OF STOMATOLOGY.

A regular meeting of the Academy of Stomatology was held on the evening of October 25, 1898, at 1731 Chestnut Street, with the President, Professor M. H. Cryer, in the chair.

An extempore lecture on "Porcelain Inlays as made by Dr. Jenkins, of Dresden," was delivered by Edwin T. Darby, M.D., D.D.S.

(For Professor Darby's lecture, which was stenographically reported, see page 73.)

### DISCUSSION.

*Dr. Deems* (Baltimore).—I do not know that I can say anything very much to add to what Professor Darby has said. I had the pleasure of making about two hundred of these inlays, and some of them are very perfect operations. There are some essential points. It is necessary to have a cavity shaped at first so that there is no undercut whatever. It is not necessary to use wax or any adhesive material in order to get the impression out. The best way to remove the impression from a cavity is gradually to coax it with a very fine instrument, inserted beneath the edge, and it will suddenly jump out of itself without being altered in shape. Another essential is perfect cleanliness in handling the material; to secure this the slab upon which the material is mixed should be covered all the time, as the least dust will very likely rise through the body and make a bubble on the surface of the inlay. The fusion of the body must be accomplished slowly, and when the body begins to fuse it should be kept at the fusing-point until seen to flow perfectly. The entire mass will flow at once, just as though one were melting a globule of gold. The retention of the inlay in the cavities is entirely dependent on the cement, and with a diamond disk, three-eighths of an inch in diameter, a number of undercuts should be made in the inlay at points corresponding to undercuts made in the cavity. A disk of small diameter assures a decidedly more steady motion than a larger one. The porcelain, when ground, appears to have the consistence of an Ash tooth, though it is not as strong. The fusing-point of the body is just below that of pure gold, and decidedly higher than that of the Downie body, and in melting it one is not troubled with bubbles, as in the case of the Downie body.

(Replying to questions by Drs. Truman and Guilford.) There is no danger at all of melting the gold matrix which is used, because it is impossible to get platinum thin enough, and, besides, the platinum cannot be adapted so closely to the margins of the cavity. The cement might perhaps wash out to a depth of a hundredth of an inch, which is practically nothing. The proper thickness of gold to use is No. 30 or No. 40.

*Dr. Roberts.*—In getting the contour of a molar or bicuspid proximal cavity, is it possible to build out that contour without having the body fuse in such a manner as to leave a round corner instead of a sharp contour. With glass inlays it is impossible to do it. Again, when glass is ground and polished, it turns black as coal in the mouth. I have never seen one that did not become black, and would like to know whether this body of Dr. Jenkins is sufficiently dense to enable it to be polished and not get black.

*Dr. Darby.*—It does not turn black, for the reason that it is superior to the glass inlay in density.

*Dr. Deems.*—I have never seen any turn black, at least none of those I have made in the last three or four years, but I have seen glass inlays in that condition, and I have seen the Downie body black, with bubbles upon the surface, and disintegrated at the cervical portion of the filling. I have several specimens of sharp contours which I shall be pleased to show.

(Replying to Dr. Inglis.) It would be possible to use the ordinary gas furnace or an electric furnace, or one need not have a furnace. You can take some of Teaque's compound, make a little muffle, place it over charcoal, and apply a blow-pipe flame to the bottom of the muffle.

*Dr. Roberts.*—A small disk of thin copper used with water or oil will cut as perfectly as a diamond disk, and even more quickly.

At this point Dr. Darby gave a demonstration, making a porcelain inlay to fit a previously prepared cavity, and the specimens of Dr. Deems were exhibited.

*Dr. McQuillen.*—I cannot add much to what Dr. Darby has said, but I have enjoyed seeing the work very much. I am still experimenting with this body, though I have not, so far, made any inlays for practical use, but am intensely interested in this kind of work. Dr. Jenkins suggested, the last time I heard from him, the necessity for thoroughly polishing the edges of the cavity before taking an impression.

*Dr. Inglis.*—I know nothing at all about the porcelain Dr. Darby has shown us. The work is certainly very beautiful, and the specimens that have been shown are quite perfect. From its appearance the porcelain seems to have a density even greater than that which characterizes the Land body. My experience with porcelain has been entirely with the Land and Moffitt bodies, but I have had some experience with German glass and the Timme glass body. I found the latter two entirely worthless and without stability as filling-materials. I was enabled to get good color, because of the opacity of the glass, and the fillings did not show good edges. The Land and Moffitt bodies occasionally give a frail edge, but when used in bulk, as in contours, it gives a very strong edge. The color of the teeth can be matched to a nicety. I have been very much pleased with this demonstration. The apparatus shown is beautifully adapted to the work, but I believe we can use this porcelain in the ordinary furnaces in use at the present time, such as the Land and Downie gas-furnaces and the electric oven.

*Dr. Guilford.*—Mr. President, I have not had any experience with porcelain inlays, but have had with glass inlays. I have placed very few in the mouth, and these were not satisfactory. I made quite a number of demonstrations before the students, simply to show them how the matrix was shaped or formed to the cavity, how the glass was melted, and so on, but in so doing I obtained quite a little experience in regard to thè preliminaries of the work.

There are some points that Dr. Darby did not touch upon. First of all, in regard to using the metal matrix, the credit for that belongs to Dr. Land, of Detroit. He had a patent on the method, and made the first real porcelain fillings that I know of that were baked. Before that inlays were cut from artificial teeth.

It was said to-night that gold is the best material for forming the matrix, but platinum can be shaped equally well and cannot be melted. A good way of forming the matrix is to get it into reasonable shape, as well as you can, without tearing it, drop some hard wax into it, and then with a warm burnisher press it in every direction. When it is removed you have a perfect mould of the cavity.

*Dr. Darby.*—There is no danger of gold melting.

*Dr. Guilford.*—I have seen it melt in making glass inlays. If gold will answer the purpose, of course, it is very well. Perhaps you will remember when Dr. Herbst was in this country, he showed us his way of preparing the cavity without undercuts, taking two modelling compound impressions of the cavity, and then with those impressions he made moulds of sand and plaster, just as we would for investing. In the one he put his tooth body, and with the blow-pipe and a little muffle fused it. Then he took it out of that one and placed it in the other, with more material, and fused it again. That same idea has been utilized by Dr. Moffitt. He does not make a platinum or gold matrix, but takes an impression of the cavity with modelling composition, and from that he makes a plaster mould, and in the plaster mould he places his body, puts it in the Custer electric oven, and fuses it. The plaster does not crack in the uniform heat. To provide for the contraction of the porcelain in cooling, he enlarges his plaster mould a little before baking the inlay. My plan of providing for the shrinkage is to give the modelling compound impression two or more coats of thick shellac varnish. This enlarges it sufficiently and evenly throughout its entire extent. By regulating the thickness of your varnish and the number of coats you will be able to get the mould just right. The specimens shown here to-night are very beautiful, and yet we know that porcelain contracts about one-fifth of its bulk, or a little less, in baking,—all porcelain does,—and if you have the thickness of the gold to take off in addition, it would seem to make a great deal of difference between the size of the inlay and that of the cavity. In the specimens that the doctor brought from Baltimore I did not see that difference exist. I do not know how to account for it, but if we could do away with the making of the metal matrix I think it would be a great advantage. I propose trying the Jenkins body in the Custer furnace.

*Dr. Inglis.*—In using the platinum matrix without investment the first shrinkage of porcelain alters the shape of the matrix. It should be replaced in the cavity and reburnished. The second baking does not seem to cause any notable warpage, nor does the third. I would like to ask Dr. Deems if there is any change in the shape of the gold matrix when invested in asbestos?

*Dr. Deems.*—I do not think there is any change of shape, because a little asbestos is placed over the edges; but the reason the inlay fits so closely is that the gold is thin and can be burnished so much more perfectly than platinum.

Adjourned.

Otto E. Inglis,
*Editor Academy of Stomatology.*

