# Correction

**Published:** 1899-10

**Authors:** 


### CORRECTION.

On page 116 of the February issue of this journal is a report of Dr. Howard E. Roberts's remarks at the Academy of Stomatology. He is made to say, "A small disk of thin copper used with water or oil will cut as perfectly as a diamond disk." This should have been, "water or oil and fine emery."

